# Production of *Trametes* *pubescens* Laccase under Submerged and Semi-Solid Culture Conditions on Agro-Industrial Wastes

**DOI:** 10.1371/journal.pone.0073721

**Published:** 2013-09-03

**Authors:** Juan C. Gonzalez, Sandra C. Medina, Alexander Rodriguez, Johann F. Osma, Carlos J. Alméciga-Díaz, Oscar F. Sánchez

**Affiliations:** 1 Chemical Engineering Department, Universidad de los Andes, Bogotá, Colombia; 2 Proteins Expression and Purification Laboratory, Institute for the Study of Inborn Errors of Metabolism, Pontificia Universidad Javeriana, Bogotá, Colombia; 3 CMUA, Department of Electrical and Electronics Engineering, Universidad de los Andes, Bogotá, Colombia; Missouri University of Science and Technology, United States of America

## Abstract

Laccases are copper-containing enzymes involved in the degradation of lignocellulosic materials and used in the treatment of phenol-containing wastewater. In this study we investigated the effect of culture conditions, i.e. submerged or semi-solid, and copper supplementation on laccase production by *Trametes* *pubescens* grown on coffee husk, soybean pod husk, or cedar sawdust. The highest specific laccase activity was achieved when the culture was conducted under submerged conditions supplemented with copper (5 mM), and using coffee husk as substrate. The crude extracts presented two laccase isoforms with molecular mass of 120 (Lac1) and 60 kDa (Lac2). Regardless of the substrate, enzymatic crude extract and purified fractions behaved similarly at different temperatures and pHs, most of them presented the maximum activity at 55 °C and a pH range between 2 and 3. In addition, they showed similar stability and electro-chemical properties. At optimal culture conditions laccase activity was 7.69±0.28 U mg^-1^ of protein for the crude extract, and 0.08±0.001 and 2.86±0.05 U mg^-1^ of protein for Lac1 and Lac2, respectively. In summary, these results show the potential of coffee husk as an important and economical growth medium to produce laccase, offering a new alternative use for this common agro-industrial byproduct.

## Introduction

Lignin, cellulose, and hemicellulose are the major compounds present in plant residues. Among them, cellulose and hemicellulose can be decomposed by a large number of aerobic and anaerobic microorganisms through the action of hydrolytic enzymes [1]. Conversely, lignin biodegradation occurs at a lower rate than plant cell wall polysaccharides [2]. Certain fungi, mostly belonging to Basidiomycetes, such as white- and brown-rot fungi, are known to be able to degrade lignin from lignocellulosic biomass [3]. The extracellular enzymatic system responsible for lignin degradation consists of lignin peroxidase (LiP, E.C. 1.11.1.14), manganese-dependent peroxidase (MnP, E.C. 1.11.1.13), and laccase (para-benzene-diol: oxygen oxidoreductase, EC 1.10.3.2I) [2,3]. While LiP catalyzes the oxidation of non-phenolic aromatic compounds such as veratryl alcohol, and MnP mainly oxidizes phenolic compounds [2], laccase catalyzes the oxidation of phenolic substrates with the concomitant reduction of oxygen to water [4]. However, the number of substrates that laccase can oxidize might be extended by using low molecular mass mediators (i.e. hydroxybenzotriazole, violuric acid, 2,2',6,6'-tetramethylpiperidine-1-oxyl), which are oxidized by laccase to organic radicals intermediates that in turn act as redox mediators [5]. In general, laccases are glycosylated and copper-containing enzymes with a molecular mass between 60 and 80 kDa, and an isoelectric point (pI) between 3.0 to 6.0 [6].

The role of laccases in lignin and phenolic compound degradation has been evaluated in a large number of biotechnological applications such as dye degradation, bioremediation of some toxic chemical wastes (e.g. chlorinated aromatic compounds, polycyclic aromatic hydrocarbons, nitroaromatics, and pesticides), and biosensor developments [3,7]. In addition, laccases have been used in food industry for wine and beer stabilization, fruit juice processing, and different food-related biosensors [8].

Use of industrial and agricultural wastes for laccase production by white-rot fungi is an effective way to reduce the cost of production [9,10]. In addition, laccase-mediated delignification allows to increase the nutritional value of agro-industrial byproducts for animal feed or soil fertilizer. To accomplish the transformation of these agro-industrial wastes, submerged and solid state cultures have been conducted (Table 1). Submerged fermentations are commonly used in industrial process; although they can present drawbacks due to physical space, and energetic and water requirements [11]. On the other hand, solid state cultures present several advantages like mimicking the natural habitat in which the microorganism grows, reduced water activity that reduce microbial contaminations, and limited water consumption and equipment size. Also, it has been reported that solid-state cultures present higher volumetric yield, less energy requirements, and higher end-product stability and concentration than submerged cultures [11,12]. Nevertheless, there are important issues related to heat and mass transfer that must be overcome in order to scale up a solid-state culture, in addition to accrued estimation of the biomass and recovery of the end product [12]. Semi-solid culture has been studied as an alternative to address some of the drawbacks presented by submerged and solid-state cultures. The increment of water activity in this type of culture allows to improve the nutrient and end-product availability, and culture control [13]. It has been established that nature and moisture of the agro-industrial waste used for a solid-state culture are critical factors [12]. The evaluation of these parameters among other process conditions, i.e. culture temperature, pH, and aeration, has encouraged the screening of several agro-industrial wastes for the production of different enzymes. In spite that several studies have been reported for the production of laccase in semi-solid culture using *Phanerochaete chrysosporium*, few studies are referred to its production in semi-solid culture using *Trametes* *pubescens* [14–16].

**Table 1:** Agro-industrial byproducts used as substrates for laccase production.

**Agro-industrial byproduct**	**Culture **	**Organism**	**Activity (U g^-1^ or UL^-1^)**	**Substrate**	**Reference**
Sugarcane bagasse	SF	*Pleurotus ostreatus*	112±2.8	ABTS	[56]
	SF	*Phanerochaete chrysosporium*	25.2±1.5	ABTS	[56]
	SF	*Pycnoporus* *cinnabarinus*	2	ABTS	[57]
	SmF	*T* *. versicolor*	410	ABTS	[58]
Coffee pulp	SF	*Ganoderma* *sp* *.*	142	ABTS	[59]
	SF	*Pleurotus ostreatus*	1	SGZ	[60]
	SF	*Pleurotus* *pulmonarius*	1.2	SGZ	[60]
Wheat bran	SF	*Pleurotus* *sp.* * IE137*	~145	SGZ	[61]
	SF	*Pleurotus* *pulmonarius* CCB19	20000	SGZ	[62]
	SmF	*Pleurotus ostreatus 1804*	8033	ABTS	[63]
	SmF	*Ganoderma* *lucidum* 447	97340±9460	ABTS	[64]
Banana peels	SF	*Ganoderma* *sp* *.*	974	ABTS	[59]
	SF	*Pleurotus* *florida*	5.4	ABTS	[65]
	SF	*Lentinula* *edodes* 122	390.31	ABTS	[66]
	SF	*Trametes* *hirsuta* BT 2566	3550	ABTS	[67]
Mandarin peels	SmF	*Ganoderma* *lucidum* 447	3145±370	ABTS	[64]
	SF	*Pleurotus* *florida*	3.1	ABTS	[65]
	SmF	*Ganoderma* *lucidum* 447	35980±3616	ABTS	[64]
	SmF	*Trametes* *pubescens*	100	ABTS	[68]
Cantaloupe peels	SmF	*Trametes* *pubescens* IBB 663	1084±92	ABTS	[67]
Soy bran	SF	*Pleurotus* *florida*	4.0	ABTS	[65]
Vinasse + Cotton stalk extract	SmF	*Ganoderma* *lucidum* 447	93840±9566	ABTS	[64]
Tomato pomace	SmF	*Coriolus* *versicolor* ATCC 200801	4406	SGZ	[69]
Grape stalks	SmF	*Funaliatrogii* ATCC 200800	4880	SGZ	[70]
Barley straw	SF	*Pleurotus ostreatus*	15	ABTS	[70]
Grape seeds	SF	*Trametes* *versicolor*	35	ABTS	[71]
	SmF	*Trametes* *versicolor*	~800	ABTS	[71]
Wheat straw	SmF	*Trametes* *versicolor*	~500	ABTS	[71]
	SmF	*Trametes* *versicolor*	~500	ABTS	[66]
Reed grass	SF	*Trametes* *hirsuta* BT 2566	22550	ABTS	[72]
Bean stalk	SF	*Lentinula* *edodes* 122	548.67	SGZ	[67]
Barley bran	SF	*Trametes* *pubescens* IBB 663	162±20	ABTS	[72]
Barley bran + Cu^+^2	SF	*Lentinula* *edodes* 122	257.69	SGZ	[66]
Apple peels	SF	*Trametes* *hirsuta* BT 2566	15740	ABTS	[67]
	SF	*Trametes* *pubescens* IBB 663	188±20*	ABTS	[67]
Tree leaves	SmF	*Trametes* *pubescens* IBB 663	1680±190	ABTS	[67]
	SF	*Trametes* *pubescens* IBB 663	280±23*	ABTS	[67]
Olive mill wastewater	SmF	*Trametes* *pubescens* IBB 663	834±61	ABTS	[67]
	SF	*Trametes* *pubescens* IBB 663	205±25*	ABTS	[67]
Wood shavings	SmF	*Trametes* *pubescens* IBB 663	630±54	ABTS	[73]
	SmF	*Panus* *tigrinus* CBS 577.79	4600±98	2,6-DMP	[73]
	SF	*Panus* *tigrinus* CBS 577.79	1309±20*	2,6-DMP	[74]
	SmF	*Trametes* *versicolor* CBS 100.29	451	ABTS	[75]
Wood shavings + Cu^+^2	SF	*Ceriporiopsissubvermispora*	~0.5	N.I.	[75]
	SF	*Ganoderma* *sp* *.*	~500	ABTS	[59]
	SF	*Trametestrogii* MYA 28-11	901	ABTS	[76]

SF: Solid state fermentation; SmF: Submerged fermentation

^*^Laccase activity expressed as UL^- 1^ for these solid state cultures

SGZ: Syringaldazine; 2,6-DMP: 2,6-dimethoxyphenol; N.I.: No indicated

Agriculture is one of the major economic activities in Colombia. Thus, there is a large production of agro-industrial byproducts, and a concern about their disposal. In this study, coffee husk, soybean pod husk, and cedar sawdust were evaluated as growth substrates for laccase production by *T* *. pubescens* CBS 696.94 under submerged (SmC) or semisolid cultures (SSC). The effect of Cu^+2^ supplementation and culture method on laccase production was also evaluated. The different crude extracts were purified, and the obtained laccase fractions were partially characterized. Results showed coffee husk as an alternative for laccase production regardless to the culture mode. This probably owe to its content of caffeine and tannins that might act as laccase inducer. In addition, the dependence of the laccase production yield and the culture mode showed to be substrate dependent.

## Materials and Methods

### Microorganism and agro-industrial substrates

Laccase was produced by using the white-rot fungus *T* *. pubescens* CBS 696.94. Soybean pod and coffee husk were obtained from Corporación *Colombiana* de Investigación Agropecuaria (CORPOICA) and a local coffee-processing company, respectively. The cedar sawdust was obtained from a local market in Bogota (Colombia). The different byproducts were treated with a 0.5% v/v hypochlorite solution for 5 h. After that, they were rinsed with distilled water until obtaining a neutral pH. Finally, the byproducts were oven dried at 60 ^°^C until constant weight. The soybean pod and coffee husk were crushed to an average particle size of about 0.8 x 0.5 mm, while cedar sawdust was sieved. The portion retained by a No. 100 sieve (opening 0.15 mm) was used for laccase production.

### Fungal culture, maintenance, and laccase production

*T* *. pubescens* was cultured on malt extract agar (MEA) plates for 10 days at 30 ^°^C. Colonized agar plugs were used for subculture maintenance every 15 days. Independent cultures, using each of the mentioned byproducts, were conducted to produce laccase under SmC or SSC conditions. Each culture was conducted in 1000 mL Erlenmeyer containing 15 g of the desired dry byproduct and 150 or 50 mL of basal medium for SmC or SSC, respectively [basal medium composition per liter: 0.5 g glucose, 2 g KH_2_PO_4_, 0.25 g MgSO_4_·7H_2_O, 0.9 g (NH_4_)_2_SO_4_, 0.1 g CaCl_2_ and 0.5 g KCl, in a citrate buffer 20 mM, pH 4.5, supplemented with 0.5 g L^-1^ thiamine [17]]. Each culture flask was inoculated with three 10-mm plugs of active fungus cultured on MEA, and incubated for 25 days at 30°C without agitation. Culture supernatant samples of 0.5 mL were withdrawn to monitor laccase activity. The effect of Cu^+2^ on laccase production was evaluated within a 0 to 5 mM concentration range (supplied as copper sulphate). The copper solution was added to 4-days-old cultures. A control was prepared by growing the fungus in the basal medium without any agro-industrial waste under the described culture conditions. All the assays were performed in triplicate.

### Laccase purification and characterization

Laccase purification and characterization was conducted using the crude extract that showed the highest enzyme activity for each substrate under the evaluated culture conditions (SmC or SSC). The crude extract was obtained by vacuum filtration through paper Whatman No. 1 followed by centrifugation at 4 °C and 4000 rpm for 15 min, serial vacuum filtration through 0.45 and 0.22 µm polyether sulphone membranes (Pall Corp, Port Washington, NY USA), and ultrafiltration (UF) through a 10 kDa cut-off membrane of regenerated cellulose (Millipore, Billerica, MA, USA). The retentate (~10 mL) was analyzed by low-pressure chromatography with a fraction collector (BioLogic^TM^, LPC, Biorad, NJ, US) using a Q-Sepharose column (Pharmacia Biotech, 1.0 x 10 cm glass chromatography column filled with 6 mL media) equilibrated with 50 mM sodium acetate buffer pH 5.0. Elution was performed by a linear NaCl gradient (~20 mL) from 0 to 0.5 M. All procedures were carried out at 4 °C. Fractions corresponding to protein peaks were collected and analyzed by sodium dodecyl sulfate-polyacrylamide electrophoresis gel (SDS-PAGE) under non-reducing conditions and without heat denaturation. Staining of the electrophoresis gel was done with 5 mM 2,2’-azino-bis(3-ethylbenzothiazoline-6) sulphonic acid (ABTS) in succinic acid (25 mM, pH 4.5) to detect laccase activity. Fractions that revealed the same protein profile and laccase activity were combined. Samples of the combine fractions were analyzed by SDS-PAGE under mentioned conditions. As a negative control were used a sample of the ultrafiltrated fraction which did not reveal laccase activity along the previous purification step. Bands that revealed laccase activity were identified as Lac1 and Lac2. These bands were excised from the electrophoresis gel and analyzed through nanoLC-MS/MS (Applied Biomics, Hayward, CA, USA). Peptides and protein sequences were aligned by using Clustal Omega [18] at EBI web server, and Blast at NCBI web server. Alignments figures were generated using CLC Sequence Viewer (CLC bio, Denmark).

Enzyme activity was determined spectroscopically by using either 2,2’-azino-bis(3-ethylbenzothiazoline-6) sulphonic acid (ABTS, ε_420_ = 36mM^-1^ cm^-1^, Sigma-Aldrich) or 4-hydroxy-3,5-dimethoxybenzaldehyde (Syringaldazine, ε_530_ = 65mM^-1^ cm^-1^, Sigma-Aldrich) [19–21]. ABTS activity assay was carried out as previously described [21,22]. Briefly, 850 µL of 5 mM ABTS in succinic acid (25 mM, pH 4.5) were mixed with 150 µL of the respective dilution of culture extract and the absorbance was at 420 nm. Syringaldazine activity assay was carried out following the substrate manufacturer’s instructions (Sigma-Aldrich). One unit (U) was defined as the amount of enzyme required to oxidize 1 µmol of substrate (ABTS or syringaldazine) per minute. Specific activity was expressed as U mg^−1^ of protein as determined by Lowry assay [23].

Laccase production during the fungus culture was monitored using syringaldazine, which has demonstrated to be a specific substrate for detecting extracellular laccase in the absence of hydrogen peroxide [24]. Absence of hydrogen peroxide was confirmed by incubation with catalase. Crude extracts and purified fractions were analyzed by SDS-PAGE without reduction by β-mercaptoethanol and heat denaturation. Proteins were visualized by silver staining, while laccases were identified by placing the SDS-PAGE gel in an ABTS solution for 5 min. Temperature and pH effect on laccase activity was determined for the UF retentate and the purified fractions by using ABTS. The temperature effect on laccase activity was evaluated at 30, 40, 50, 55, 60 and 70 °C at pH 5.0, and the pH effect on laccase activity was evaluated at 2, 2.5, 3, 4, 5, 6, 6.5 and 7 at 55 °C. Laccase stability was evaluated by using ABTS at optimal pH and temperature for 48 h.

### Cyclic voltammetry analysis

Cyclic voltammetry (CV) analysis was performed on a computer-controlled potentiostat (Autolab Potentiostat/Galvanostat PGSTAT 128N, Metrohm, USA). Data were acquired by using the software Nova version 1.6. A conventional three-electrode system was used to carry out the cyclic voltammograms. The used electrodes were graphite-working electrode with a surface area of 0.23 cm^2^, Ag/AgCl KCl saturated reference electrode (Metrohm, USA), and platinum wire counter electrode. Before running each experiment, the surface of the graphite electrode was polished with alumina and thoroughly rinsed with distilled water. CV analysis was done to the crude extract and purified laccase fractions that presented the highest activity for each substrate. For the test, 20 µL of sample were added on the polished surface of the working electrode, and allowed to dry for 60 min at room temperature. Each experiment was performed in triplicate using either a solution 1:1 of anhydride ethanol: buffer acetate (100 mM, pH 5.0) and syringaldazine to a final concentration of 0.2 mM; or a solution of 100 mM acetate buffer (pH 3.0) and 0.5 mM ABTS. Cyclic voltammograms were carried out in 30 mL of working solution with continuous agitation. Voltage was scanned at 100 mV s^-1^ between -0.2 and 1 V vs. Ag|AgCl|KCl_sat_ for syringaldazine assays while for ABTS assays voltage was scanned at 250 mV s^-1^ between -0.2 and 1.2 V vs. Ag|AgCl|KCl_sat_. Assays were conducted in a Faraday cage at room temperature (20-22°C). Temperature and pH in the reaction solution was monitored with the Unitrode with Pt 1000 (Metrohm, USA).

### Statistical analysis

All experiments were conducted by triplicate. Data are presented as the mean ± standard deviation (SD). Statistical comparisons were conducted by using either t-test or Duncan’s test for one-factor design by using StatGraphics Centurion^®^ v. 16.1.11 (2011). Statistical differences were established for *p* < 0.05.

## Results

### SmC and SSC production of laccase by T. pubescens

**Figure 1:**
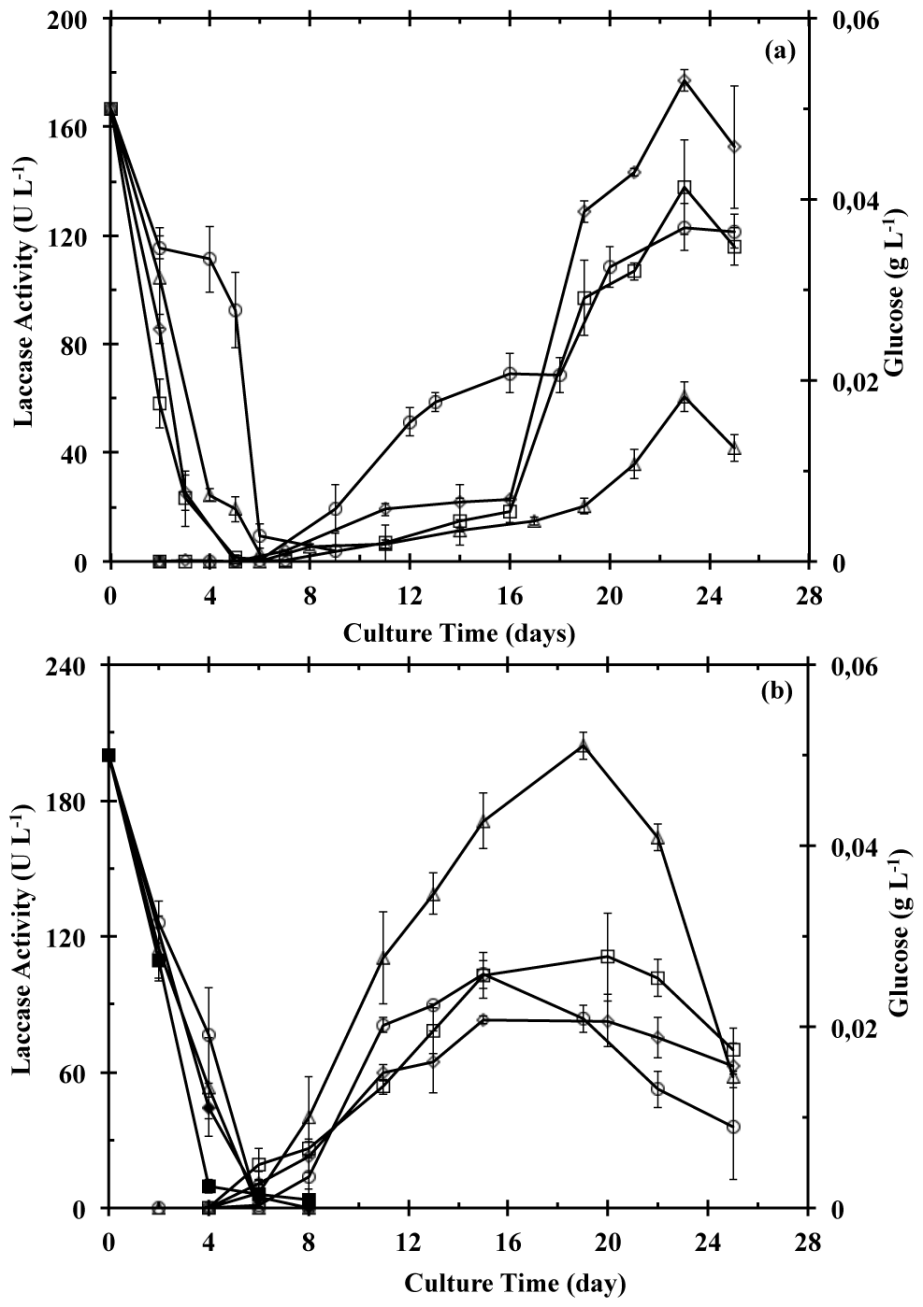
Laccase activity profiles during *T* *. pubescens* CBS 696.94 culture under SmC (a) and SSC (b) on coffee husk. Culture media was enriched with different copper concentrations: 0 (Δ), 0.5 (□), 2 (○) and 5 (◊) mM, and laccase activity was determined by using syringaldazine as substrate. Filled symbols refer to the glucose concentration at the respective copper concentration. All the assays were performed in triplicate.

Coffee husk, soybean pod husk, and cedar sawdust were used as substrate for the production of laccase by *T* *. pubescens* under SmC or SSC conditions, while syringaldazine was used to monitor laccase production during the fungus culture under the studied conditions. Hydrogen peroxide was not detected in any of the evaluated samples, which discard the interference of peroxidases in the syringaldazine assay. Laccase activity and glucose concentration time course for *T* *. pubescens* grown on coffee husk under SmC and SSC at different copper concentrations are presented in Figure 1. Although, a similar time course was observed for laccase activity and glucose concentration of soybean pod husk and cedar sawdust cultures, differences on the day of maximum lacasse activity as well as in the maximum activity value were observed among the agro-industrial byproducts (Table 2). In general, it was noticed that laccase activity was detectable around the seventh and fourth day of culture for SmC and SSC, respectively. After the eighth culture day, glucose was depleted from the culture medium and laccase activity increased sharply.

**Table 2:** Effect of substrate, copper concentration and culture mode on laccase activity (UL^-1^). Syringaldazine was used for monitoring laccase activity.

**Substrate**	**SmC**	**SSC**
Soybean Pod Husk + 0 mM Cu^+^2	4.46 ± 1.38 (23)^**^	12.55 ± 0.52 (13)^**^
Soybean Pod Husk + 0.5 mM Cu^+^2	84.93 ± 8.46 (23)^†^	20.28 ± 3.87 (21)
Soybean Pod Husk + 2 mM Cu^+^2	57.61 ± 3.70 (19)	137.85 ± 0.71 (18)^†^
Soybean Pod Husk + 5 mM Cu^+^2	44.92 ± 4.87 (23)	29.00 ± 4.07 (18)
Coffee Husk + 0 mM Cu^+^2	61.84 ± 1.76 (23)^**^	204.00 ± 5.96 (19)^**^,^†^
Coffee Husk + 0.5 mM Cu^+^2	137.63 ± 5.68 (23)	110.83 ± 9.40 (20)^a^,^b^
Coffee Husk + 2 mM Cu^+^2	123.04 ± 3.73 (23)^b^	102.78 ± 8.14 (15)^b^
Coffee Husk + 5 mM Cu^+^2	177.23 ± 3.72 (23)^†^	82.66 ± 8.82 (15)
Cedar Sawdust + 0 mM Cu^+^2	13.55 ± 0.95 (17)^*^	10.82 ± 0.53 (15)^*^
Cedar Sawdust + 0.5 mM Cu^+^2	22.72 ± 0.84 (17)	19.02 ± 0.02 (13)
Cedar Sawdust + 2 mM Cu^+^2	28.97 ± 1.27 (12)^a^	26.38 ± 0.68 (8)
Cedar Sawdust + 5 mM Cu^+^2	29.36 ± 1.53 (13) ^a,b,†^	30.73 ± 1.52 (12)^b^,^†^

Data in parenthesis refers to the day where was observed the maximum laccase activity

^*^ No Statistical Difference, ^**^ Statistical Difference. (Evaluation of substrate effect on laccase activity for both culture modes, submerged (SmC) and semisolid (SSC), no copper induction considered)

Equal letters inside one agro-industrial waste group refers to No Statistical Difference; no letters refers to Statistical Difference inside the group. (Evaluation of copper effect on laccase activity for both SmC and SSC)

^†^ Maximum laccase activity for the used agro-industrial wastes either in SmC or SSC culture.

The substrate source seemed to be the most important factor for laccase production (Table 2). *T* *. pubescens* culture on coffee husk allowed the maximum laccase activities for both SmC (177.23±3.72 U L^-1^) and SSC (204.00±5.96 U L^-1^) at copper concentrations of 5 and 0 µM, respectively. Laccase activity in SSC and SmC using coffee husk as substrate was between 1.47- and 6.63-fold higher than that observed for cultures with soybean pod husk and cedar sawdust. Laccase activity obtained in the control culture, without agro-industrial waste, presented a maximum laccase activity of 2.22 ± 0.88 U L^-1^ after 14 days of culture, while the glucose was depleted on the seventh day of culture.

Copper supplementation effect on laccase activity was evaluated for both SmC and SSC. Although in a non-linear mode, the laccase activity was increased by the presence of copper with all substrates under SmC or SSC except with coffee husks under SSC (Table 2). In addition, the required copper concentration to obtain the highest laccase activity varied depending on the used agro-industrial waste. For example, laccase production was favored in cultures with soybean pod husk at copper concentrations of 0.5 mM and 2.0 mM for SmC and SSC, respectively, while for cultures with coffee husk the highest laccase activity levels for SmC and SSC was observed at copper concentrations of 5 mM and 0 mM, respectively.

Despite that culture mode, i.e. SmC or SSC, presented an effect on laccase production, this is substrate sensitive. In the assays conducted without copper, it was observed that laccase activity was 2.8- and 3.3-fold higher in SSC than in SmC for the soybean pod husk and coffee husk, respectively. However, when cedar sawdust was used, no significant difference in laccase activity between the two culture types was obtained (Table 2). Due to the low volume of liquid media and high laccase activity in SSC when compared to SmC, the SSC presented a higher volumetric yield. For all agro-industrial waste, the SSC presented a shorter time than SmC to reach the highest laccase activity, which suggest a higher productivity under SSC conditions.

These results show that coffee husk under SmC or SSC conditions could be considered as a potential alternative for laccase production by *T* *. pubescens* . However, soybean pod husk and cedar sawdust could be also considered as important alternatives for laccase production, especially in comparison with synthetic culture media [10]. Finally, the optimal copper concentration to obtain the highest laccase activity depends on the agro-industrial byproduct used for the fungus culture.

### Laccase purification and characterization

**Figure 2:**
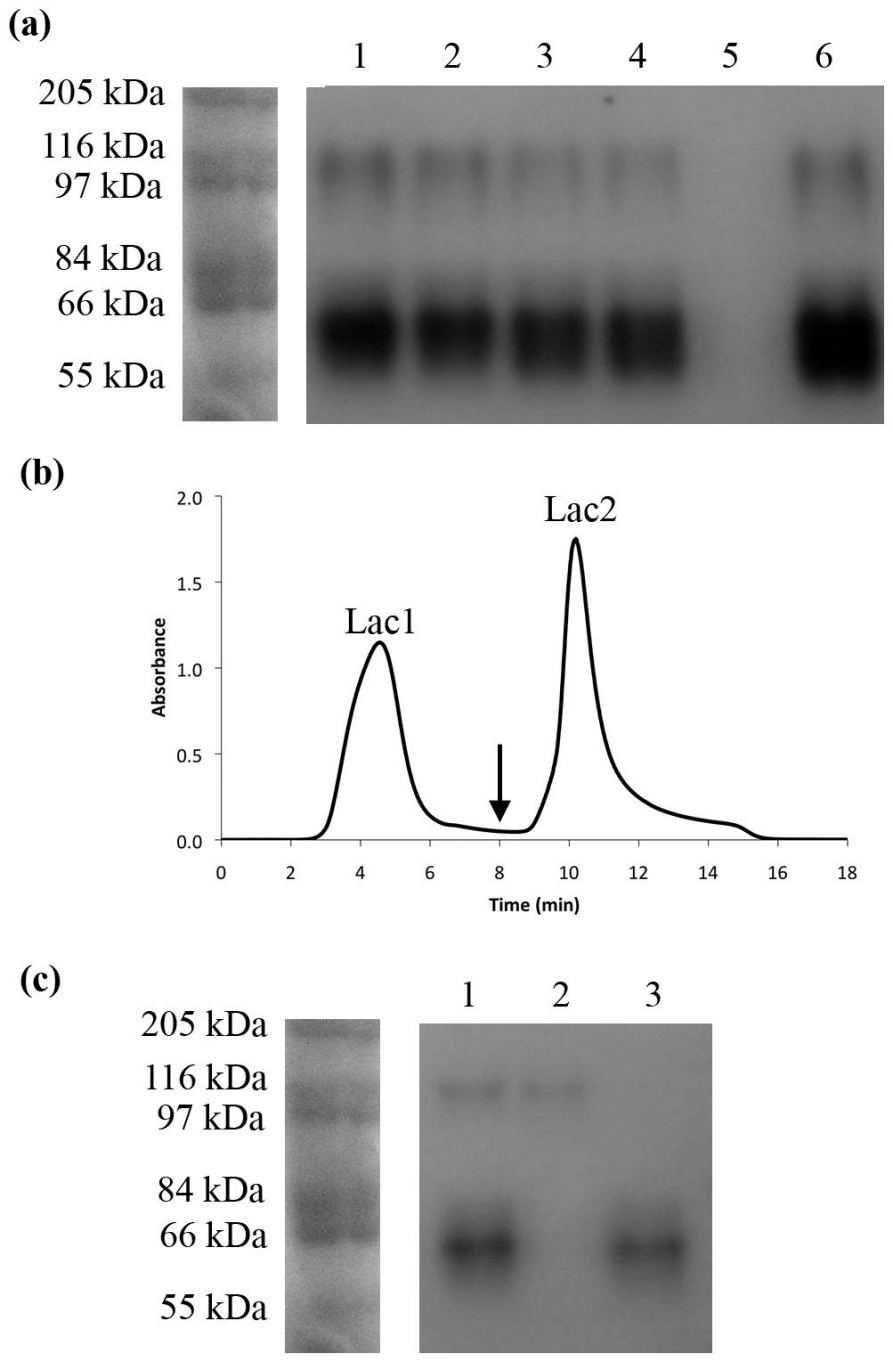
Purification of lacasses produced by submerged culture of *T* *. pubescens* using coffee husk as substrate. All samples were analyzed by SDS-PAGE under non-reducing conditions and stained with ABTS. No heat denaturation of the samples was conducted. (**a**) The crude extract (1) was centrifuged and filtered through a Whatman No. 1 filter (2), 0.45 µM membrane (3) 0.22 µm membrane (4), and ultrafiltrated through a 10 kDa cut-off membrane, permeate (5) and retentate (6). (**b**) UF retentate was purified by anionic exchange chromatography. Lac1 represent the unbound fraction, while Lac2 represent the eluted fraction. The arrow shows the point when protein elution was started. (**c**) Purified fractions (1) crude extract, (2) Lac1 and (3) Lac2. MW: Molecular Weight. Similar results were observed for crude extracts produced by using soybean pod husk and cedar sawdust.

Laccase purification was conducted using the crude extracts that presented the highest activities. For the SmC, crude extracts obtained with soybean pod husk + Cu^+2^ 0.5 mM, coffee husk + Cu^+2^ 5 mM, and cedar sawdust + Cu^+2^ 5 mM were selected; while for SSC crude extracts obtained with soybean pod husk + Cu^+2^ 2 mM, coffee husk + Cu^+2^ 0 mM, and cedar sawdust + Cu^+2^ 5 mM were selected. The electrophoretic analysis of permeates from 0.45 and 0.22 µm filtrations and the UF retentate showed the presence of two proteins with laccase activity, while no activity was observed within the ultrafiltrated sample (Figure 2a). These proteins presented a molecular mass of about 120 kDa and 60 kDa (Figure 2a). The purification of the UF retentate by ionic exchange chromatography confirmed the presence of two different fractions with laccase activity (Figure 2b). The SDS-PAGE analysis of these fractions, under non-reducing conditions, showed that one protein with laccase activity was presented on each fraction (Figure 2b), and were named Lac1 (120 kDa) and Lac2 (60 kDa). Considering that the conditions used to measure the enzyme activity were those suggested for laccase activity, and that H_2_O_2_ or manganese were not added during the reaction for measuring the enzyme activity, these results suggest that both proteins are laccases but not peroxidases.

**Figure 3:**
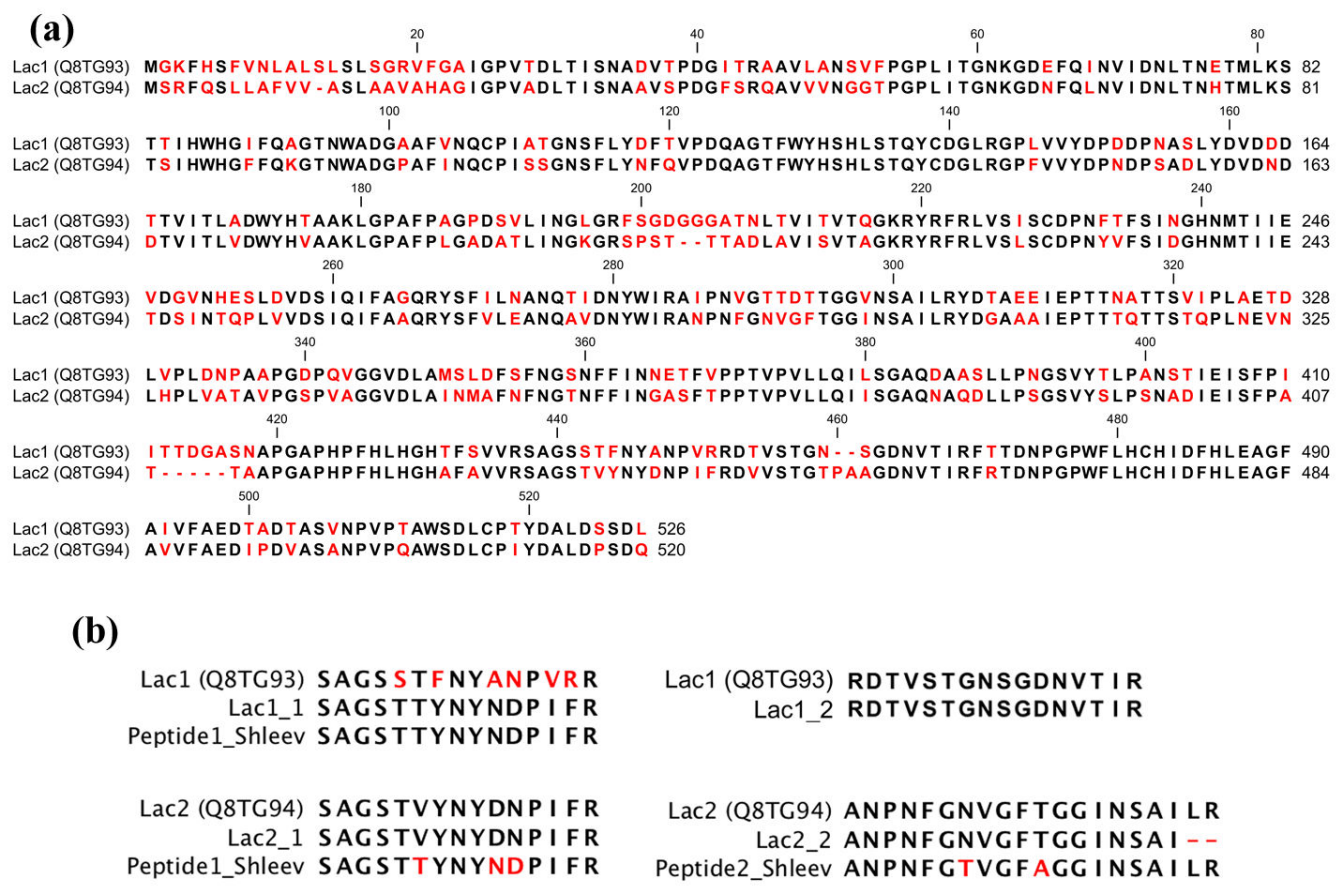
Aligment of reported lacasses sequences and Lac1 and Lac2 peptides. (a) Alignment of Lap1 (UniProtKB/Swiss-Prot Q8TG93) and Lap2 (UniProtKB/Swiss-Prot Q8TG94) from *T* *. pubescens* CBS 696.94. (b) Alignment of Lac1 and Lac2 peptides with Lap1 and Lap2, respectively, and tryptic peptides described by Shleev et al 2007. Alignments were carried out using Clustal Omega and figures were using CLC Sequence Viewer. Different residues are colored in red.

NanoLC-MS/MS analysis of purified fractions showed that the identified peptides for Lac1 (SAGSTTYNYNDPIFR and RDTVSTGNSGDNVTIR) and Lac2 (SAGSTVYNYDNPIFR and ANPNFGNVGFTGGINSAI), had identity scores between 80 to 100% with laccases (over 100 sequences) available at the non-redundant GenBank dataset. Proteins containing these peptides, different that laccases, were not identified. Similar results were observed when peptides were evaluated at Uniprot. Galhaup et al. [17] described two laccase active fractions from *T* *. pubescens* MB 89 (CBS 696.94): Lap1 (UniProtKB/TrEMBL Q8TG93) and Lap2 (UniProtKB/TrEMBL Q8TG94), which share a 70% identity (Figure 3a). Sequence alignment of Lac1 and Lac2 peptides with Lap1 and Lap2 sequences, respectively, showed that the peptides are presented within *T* *. pubescens* MB 89 laccase sequences, though some differences were observed for the Lac1 peptide (Figure 3b). This result suggests that the purified fractions are laccase isoenzymes.

Protein concentration and volumetric and specific laccase activities at different purification stages are presented in Table 3. Lac1 presented lower laccase activity than Lac2 in a ratio that ranges from 0.80- to 38-fold depending on the substrate source. On the other hand, laccase activity of the UF retentate from soybean pod husk culture was from 4.5- to 6.4-fold higher than that of the purified fractions, while for cedar sawdust and coffee husk the laccase activity was from 4.6- to 12.0-fold and from 3.3- to 163-fold higher than the purified fractions, respectively (Table 3). The purified Lac2 fractions from coffee husk and cedar sawdust cultures showed the highest specific activity levels (Table 3). The volumetric activities of UF retentates were higher than that obtained with purified fractions (Table 4).

**Table 3:** Laccase activity during the purification of crude extracts resulted from *T* *. pubescens* culture on agro-industrial wastes under submerged (SmC) and semisolid (SSC) cultures.

	**Soybean pod husk**				**Coffee husk**				**Cedar sawdust**			
	**Protein (mg mL^-1^)**	**Volumetric Activity (UL^-1^)**	**Specific Activity (U mg^-1^ protein)**	**Recovery (%)**	**Protein (mg mL^-1^)**	**Volumetric Activity (UL^-1^)**	**Specific Activity (U mg^-1^ protein)**	**Recovery (%)**	**Protein (mg mL^-1^)**	**Volumetric Activity (UL^-1^)**	**Specific Activity (U mg^-1^ protein)**	**Recover (%)**
	**SmC**											
**Crude Extract**	0.869	528.88	0.608	100	0.414	1527.08	3.688	100	0.431	107.35	0.249	100
**0.45 µm Filtration**	0.736	465.49	0.632	88	0.334	1178.92	3.529	77	0.444	120.53	0.271	112
**UF retentate**	1.010	787.31	**0.779**	74	0.378	1952.63	5.165	64	0.510	259.05	0.507	120
**Lac1**	0.494	54.71	0.110	1	0.232	23.99	0.103	0.2	0.222	8.38	0.037	0.8
**Lac2**	0.310	206.61	0.666	4	0.116	1072.40	**9.252**	7	0.040	152.34	**3.837**	14
	**SSC**											
**Crude Extract**	1.146	1649.66	1.439	100	0.596	1549.07	2.601	100	1.065	232.03	0.217	100
**0.45 µm Filtration**	0.776	1283.92	1.654	77	0.474	1157.36	2.439	75	0.806	258.24	0.320	111
**UF retentate**	0.973	3350.55	**3.443**	101	0.428	1617.50	3.779	52	1.134	511.01	0.450	110
**Lac1**	0.097	88.54	0.912	2	0.104	15.14	0.145	0.3	0.042	13.15	0.313	2
**Lac2**	0.168	489.88	2.915	10	0.131	822.98	**6.282**	18	0.072	161.62	**2.244**	23

740 ABTS was used to determine laccase activity.

**Table 4:** Optimum temperatures and pHs for maximum laccase activities in UF retentate, Lac1 and Lac2 obtained from semisolid cultured *T* *. pubescens* on agro-industrial wastes.

**Enzymatic Fraction**	**Temperature**		**pH**	
	**Volumetric Activity (UL^-1^)**	**Specific Activity (U mg^-1^ protein)**	**Volumetric Activity (UL^-1^)**	**Specific Activity (U mg^-1^ protein)**
Soybean Pod Husk + 2 mM Cu^+^2	1480.00±11.78 (55)	1.521±0.012	1820.37±30.25 (2-3)	1.871±0.031
Lac1	229.25±9.17 (60)	2.363±0.095	361.35±8.31 (2,3)	3.725±0.085
Lac2	326.38±15.58 (55)	1.942±0.092	287.13±2.74 (2,3)	1.709±0.016
Coffee Husk + 0 mM Cu^+^2	1137.40±82.89 (50-55)	3.008±0.219	2906.66±10.39 (2-3)	7.689±0.275
Lac1	8.89±0.39 (55)	0.038±0.002	17.79±0.28 (2,3)	0.077±0.001
Lac2	341.94±27.89 (60)	2.947±0.240	331.50±5.26 (2–4)	2.857±0.045
Cedar Sawdust + 5 mM Cu^+^2	2576.66±12.01 (55)	5.052±0.024	3446.34±104.3 (2-3)	6.757±0.204
Lac1	213.70±4.72 (55)	0.962±0.021	291.17±9.43 (2,3)	1.311±0.042
Lac2	551.66±16.56 (60)	13.79±0.414	551.66±16.56 (5)	13.79±0.414

ABTS was used to determine laccase activity.

Data in parenthesis shows the temperature or pH at which relative laccase activity reached 100%.

**Figure 4:**
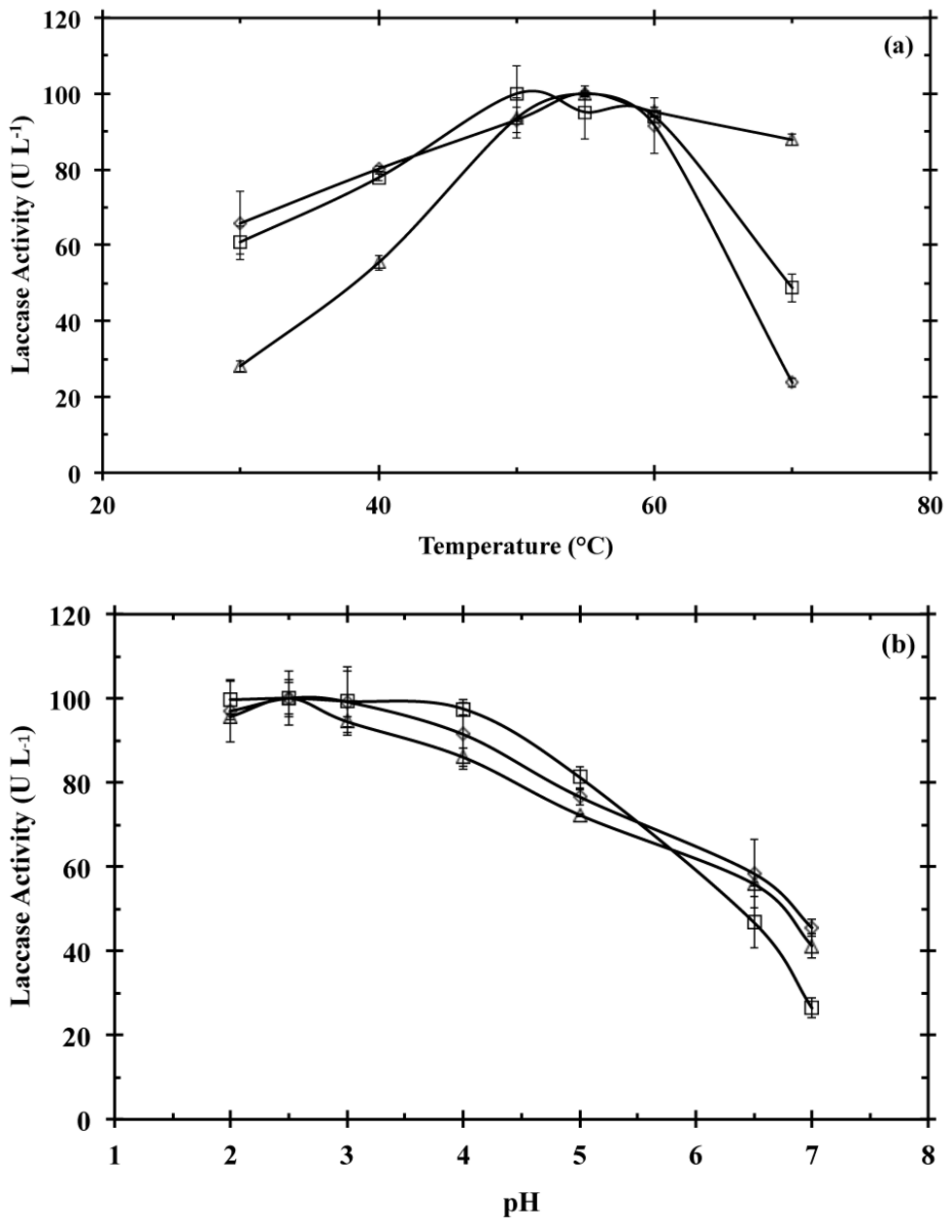
Effect of temperature (a) and pH (b) on laccase activity for the UF retentate obtained by semisolid culture of *T* *. pubescens* on coffee husk (□), soybean pod husk (◊), and cedar sawdust (Δ). Laccase stability was evaluated by using ABTS. All the assays were performed in triplicate.

Temperature and pH effect on laccase activity was determined for the UF retentate, and Lac1 and Lac2 fractions obtained along the purification of the enzymatic crude extracts that resulted after the SSC of *T* *. pubescens* on the selected agro-industrial wastes. Laccase activity for the UF retentate under different temperature and pH conditions is presented in Figure 4 and Table 4. The maximum relative activity of the evaluated UF retentates was observed at 50-55 °C, and at a pH range from 2.0 to 4.0. However, UF retentate from cedar sawdust culture behaved different to the UF retentate from coffee husk and soybean pod husk cultures. In this sense, the UF retentate from cedar sawdust culture presented the lowest enzyme activity at temperatures ≤ 40 °C, but at 70 °C presented the highest enzyme activity, ~90% of its initial value, in comparison to the other two UF retentates (Figure 4).

**Figure 5:**
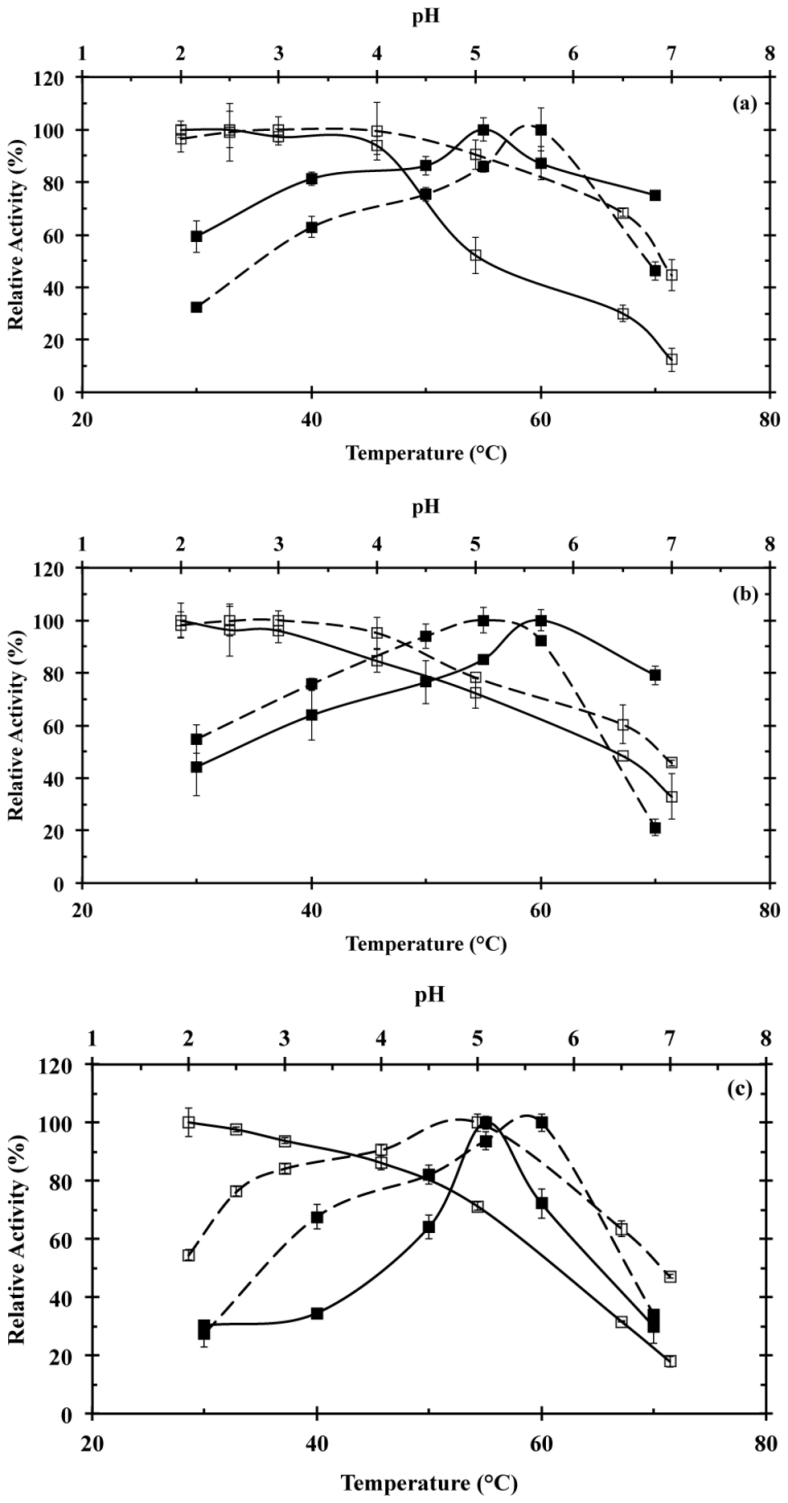
Effect of temperature (■) and pH (□) on enzyme activity for Lac1 (solid line) and Lac2 (dashed line), obtained after the purification of the enzymatic crude extract produced by semisolid culture of *T* *. pubescens* on coffee husk (a), soybean pod husk (b) and cedar sawdust (c). Laccase stability was evaluated by using ABTS. All the assays were performed in triplicate.

The temperature and pH effect on laccase activity for Lac1 and Lac2 is presented in Figure 5. The maximum relative activity for both fractions was presented at a pH from 2.0 to 4.0, regardless of the agro-industrial waste, as observed for the UF retentate. However, at pH > 4.0 Lac1 showed a sharper decrease in its activity than that observed for Lac2. Nevertheless, Lac2 purified from cedar sawdust culture presented a different performance. This fraction showed the maximum activity at pH 5.0, while a sharp drop was observed at pHs above or below this value.

Laccase activity profiles showed that Lac1 and Lac2 presented the maximum enzyme activity at 55 and 60 °C, respectively (Table 4). It was observed that Lac1 from coffee husk culture presented a lower decrease in enzyme activity at temperatures ≤ 50 °C or ≥ 60 °C than Lac2. On the contrary, Lac2 from soybean husk or cedar sawdust cultures presented a higher relative laccase activity than Lac1 at temperatures ≤ 55 °C. The purified fractions from soybean pod husk and cedar sawdust cultures presented the lowest relative activity at 30 °C and 65 °C.

### Laccase stability

For selected enzymatic extracts and purified fractions (Table 4) a stability test for 48 h at their optimal pH and temperature was conducted. Time course of the relative laccase activity for crude extract and purified fractions from coffee husk and cedar sawdust cultures are presented in Figure 5. Similar results were observed for crude extract and purified fractions from soybean pod husk culture (data no shown).

**Figure 6:**
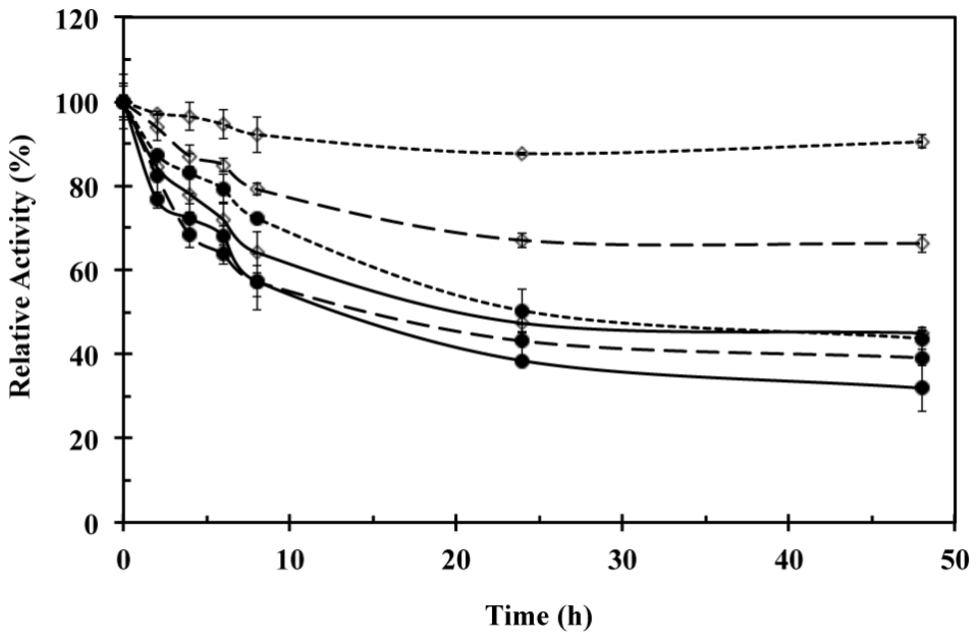
Laccase stability profiles at optimal pH and temperature conditions for crude extracts (solid line), Lac1 (dashed line), and Lac2 (spotted line) obtained from semisolid culture of *T* *. pubescens* on coffee husk (◊) and cedar sawdust (•). Laccase stability was evaluated by using ABTS. All the assays were performed in triplicate.

It was observed that the activity loss depends on the substrate source used to grow *T* *. pubescens* . An activity loss of 55% was observed for crude extracts and purified fractions from coffee husk and soybean pod husk cultures, while an activity loss of 70% was observed for the crude extract and purified fractions from cedar sawdust culture. Nevertheless, Lac2 showed the lowest activity reduction, with values of 10, 40 and 55% for coffee husk, soybean pod husk, and cedar sawdust, respectively, while Lac1 presented an activity loss of 34, 50 and 60% for coffee husk, soybean pod husk, and cedar sawdust, respectively. Despite the differences in laccase activity among agro-industrial wastes and between crude extracts and purified fractions, all the profiles showed a sharp decrease during the first 8 h while a reduced activity loss was observed during the last 24 h of the assay (Figure 6).

### Laccase electrochemical characterization

**Figure 7:**
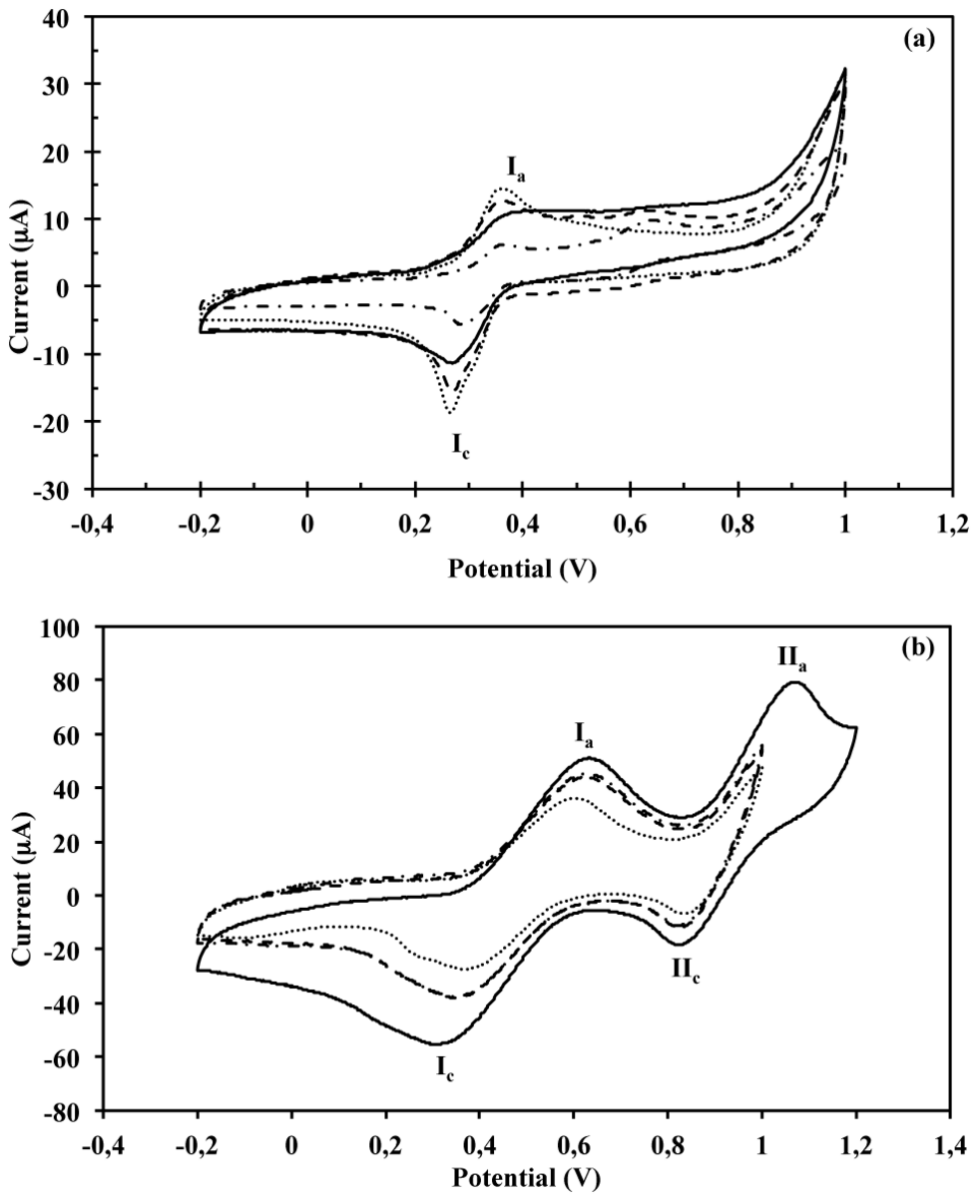
Cyclic voltammograms of (a) syringaldazine at pH 5.0 using the crude extract and purified laccase fractions from semisolid culture of *T* *. pubescens* on coffee husk, or (b) ABTS at pH 3.0 using the crude extract and purified laccase fractions from semisolid culture of *T* *. pubescens* on soybean pod husk. Nomenclature: only mediator (dotted line), crude extract (solid line), Lac1 fraction (dashed line), and Lac2 fraction (dashed and dotted line).

For selected crude extracts and purified fractions a cyclic voltammetry characterization was conducted. For each mediator, syringaldazine or ABTS, similar cyclic voltammograms were obtained for the different crude extracts and purified fractions. Voltammograms of the redox reaction for syringaldazine (Figure 7a) and ABTS (Figure 6b) are presented. Two anionic and cationic peaks were observed when syringaldazine was used as mediator. The first pair of peaks showed a reversible performance, with a mean Ia: Ic ratio of 1.09±0.06, 1.06±0.05, and 0.98±0.02, for the crude extracts, Lac1, and Lac2, respectively. The second pair of peaks showed quasi-reversible performance, and the cathodic peak was presented at a potential of +0.62 V (vs Ag|AgCl), while the anodic peak was presented at a potential of +0.60 V (vs Ag|AgCl). No significant difference on the reduction potential (E^o^) for syringaldazine was observed for the crude extracts or the purified fractions, obtaining an E^o,^ of 0.314±0.008 V (vs Ag|AgCl). However, the peak potential separation (ΔE_p_) showed a different profile depending on the substrate used for the culture. While not significant difference (*p* > 0.05) on ΔE_p_ value was observed between crude extract (0.045±0.005 V vs Ag|AgCl), Lac1 (0.046±0.005 V vs Ag|AgCl), and Lac2 (0.028±0.001 V vs Ag|AgCl), from cultures with cedar sawdust and soybean pod husk; the ΔE_p_ was considerably higher for the crude extract (0.083±0.004 V vs Ag|AgCl), Lac1 (0.068±0.003 V vs Ag|AgCl) and Lac2 (0.051±0.005 V vs Ag|AgCl) from the coffee husk culture.

For reactions conducted with ABTS and the different crude extracts and purified fractions, the voltammograms exhibited one pair of cathodic and anodic peaks (Figure 7b), which corresponds to the redox of ABTS/ABTS^+^ (I_a_/I_c_) with an E^o,^ of 0.487±0.006 V (vs Ag|AgCl). This process was reversible presenting a mean Ia: Ic ratio of 0.99±0.02. In addition, a second cathodic peak was presented at +0.83 V (vs Ag|AgCl) when ABTS redox reaction was conducted using the crude extract and the purified fractions. This performance agrees with previous reports in which the laccase activity cannot be observed at potential values of the second reversible oxidation of ABTS, ABTS^+^/ABTS^2+^, due to this catalytic process cannot be detected by cyclic voltammetry [25,26]. Nevertheless, voltammograms for laccase crude extract from a soybean pod husk culture presented the second anodic peak, which in addition to the second cathodic peak corresponds to the redox of ABTS^+^/ABTS^2+^ (II_a_/II_c_) with an E^o,^ of 0.942±0.004 V (vs Ag|AgCl). Contrary to the reactions conducted using syringaldazine as substrate, the ΔE_p_ did not present a significant difference for the crude extracts and purified fractions, 0.248±0.016 V (vs Ag|AgCl).

## Discussion

In this study, we evaluated the effect of the culture mode, i.e SmC and SSC, on laccase production by *T* *. pubescens* CBS 696.94 using three different agro-industrial wastes (coffee husk, soybean pod husk, and cedar sawdust). Furthermore, the effect of Cu^+2^ supplementation on laccase production was also evaluated, and the crude extracts and the purified fractions were partially characterized.

Substrate source for laccase production showed to be one of the most important factors for laccase production (Table 2). Cultures with coffee husk as substrate presented the highest laccase production levels. The observed differences in laccase activity for each substrate could be owed to their chemical composition. *Coffea* *arabica* is the coffee variety raised in Colombia, and its husk has a reported composition of 23.08% cellulose (20.76% glucose and 1.83% cellobiose), 23.85% hemicellulose (13.56% xylose, 5.23% arabinose, 2.56% acetic acid and 1.95% glucuronic acid); 28.28% total lignin, and 0.71% ashes [27]. In addition, coffee husk contents 1.3% caffeine, 4.5% tannins, and 12.4% pectins [28]. On the other hand, *Glycine max* is the soybean species cultured in Colombia, and its pod husk composition varies from 5.04 to 6.2% crude protein, 6.08 to 14.3% hemicellulose, 37.15 to 42.08% acid detergent fiber, 44.36 to 60.15% neutral detergent fiber, 8.50 to 8.93% acid detergent lignin, and 20.74 to 21.06% uronic acid [29–31]. Finally, for cedar sawdust the major components are glucose (42.6-44.4%), xylose (4.6-19.5%), cellulose (38.3-41.0%), klason lignin (22.7-27.8%), and acid lignin (4.0-4.3%) [32–34]. Although, lignin is one of the most important compounds associated with laccase production by white-rot fungi, we did not observe a clear correlation between lignin composition percentage and laccase activity. In that sense, the high laccase activity observed for the cultures that use coffee husk could be associated with its content of caffeine and tannins, which have been identified as potent inducers of laccase gene expression [17,35,36]. In fact, caffeine has been used as inducer for laccase production by *Rhizoctonia solani*, obtaining an increase of 4.1-fold in laccase activity [37]. Similar results were also reported for the production of *Coloriopsis* *gallica* laccase [38].

Laccase gene expression is mainly up regulated by Cu^2+^, although Mn^2+^, Fe^3+^, heavy metals, 2,6-dimethoxy-1,4-benzoquinone, H_2_O_2_, amphotericin B, syringic acid, tannic acid, Tween 80, soybean oil, aromatic compounds, and microclimatic changes (i.e. lower temperature and osmotic pressure) are also recognized as potent inducers [17,35,36]. In general, Cu^2+^ supplementation increased the laccase activity in both SmC and SSC. However, Cu^2+^concentration required to obtain the highest laccase activity varied depending on agro-industrial waste. These differences can be attributed to the copper presented in the agro-industrial waste and the copper availability in the medium. Copper can be found in amounts that range from 15.6 to 18.0 mg kg^-1^ of coffee husk [39], and from 2.6 to 6.7 mg kg^-1^ of soybean pod husk (or soybean hull) [39,40]. Despite that no reports indicate the copper content in untreated cedar wood, an average of 1.0 mg kg^-1^ of wood has been reported for wood used as biomass in six different Europe countries [41]. This allows to consider the cedar sawdust as the substrate with the lowest content of copper. On the other hand, copper adsorption by sawdust and coffee husk has been reported, obtaining a Langmuir based maximum adsorption capacity (Q_max_, mg g^-1^ adsorbent) of 8.45 and 7.5, respectively [42,43]. Although no adsorption studies has been reported using untreated soybean pod husk, Šćiban et al. [44] reported a Q_max_ for copper adsorption of 0.085 mmol g^-1^ adsorbent (~ 5.4 mg g^-1^) using treated soybean straws. Based on this information, it can be expected that a higher amount of exogenous copper would be required for laccase induction on cultures with cedar sawdust than with soybean pod husk and coffee husk due to its lower content of copper. In addition, due to the high copper-adsorption capacity of sawdust, the amount of available copper in the culture would be reduced allowing the laccase production at higher copper concentrations without reaching levels that might be toxic for the fungus. This fact can be observed in the SSC (Table 4). However, for the SmC this behavior is not maintained for the cultures with soybean pod husk and coffee husk (Table 4) where the maximum laccase activity was obtained with a copper concentration of 0.5 and 5 mM, respectively. This could be owed to a lower contact between the fungus and the substrate in the SmC when compared to the SSC which is close related to the transport of nutrients presented in the substrates.

Production of extracellular laccases has been reported in a large number of species of white-rot fungi strains grown on natural substrates (Table 1). Although a laccase activity profile is not clearly observed based on the agro-industrial waste, culture mode, and fungus strain, most cases in which SmC and SSC were conducted using the same substrate and fungus strain, showed a higher laccase activity under SmC conditions. In this study was observed a substrate dependent performance, where the most favorable condition to obtain the highest laccase activity was the SSC. The laccase activities obtained in the present study are comparable, and in some cases higher than those reported for *Pleurotus ostreatus*, *Phanerochaete chrysosporium*, *Pycnoporus* *cinnabarinus* , *T* *. versicolor* and *Ganoderma* *sp.* , cultured under SC and SmC conditions, and using different agro-industrial wastes (Table 1). Semi-solid cultures using different agro-industrial wastes have been reported using *T* *. versicolor* , *Phlebia* *radiata* , *T* *. pubescens* *, P. ostreatus*, and *C* *. unicolor* . Rodriguez et al. [16] screened different supports for laccase production by *T* *. versicolor* under SSC. They obtained the highest laccase activity (1200 U L^-1^) when in the culture medium was presented barley bran. In addition, they were able to obtain a laccase activity of 1700 U L^-1^ when the culture was supplemented with xylidine as an inducer. These results were obtained after 17 or 18 days of culture, and ABTS was used to determine the enzyme activity. Mäkelä et al. [45,46] have studied the expression of the genes involved in laccase production, *Pr-lac1* and *Pr-lac2*, presented in *P* *. radiata* , and evaluate laccase production in SSC using milled alder wood ( *Alnus* *incana* ) as substrate. They reported a maximum laccase activity of 3 µkat L^-1^ (~180 U L^-1^) at the 14^th^ day of culture and using syringaldazine for its detection. Osma et al. [15] have studied the morphological changes and laccase production of strains *T* *. pubescens* *, P. ostreatus,* *C* *. unicolor* and *T* *. versicolor* grown on wheat bran flakes using SSC. They obtained a maximum laccase production between 1397 and 2778 U L^-1^ after 10 and 13 days of culture. This enzyme activity is strain dependent, and ABTS was used for its quantification. Our results show a good correlation with reported data. The highest laccase activity was obtained when *T* *. pubescens* was grown on coffee husk under SSC without copper (204 U L^-1^ evaluated with syringaldazine or 1549 U L^-1^ evaluated with ABTS), which is in some cases higher than reported ones. This shows that under the studied conditions, the production of the laccase crude extract is favored in SSC using coffee husk without the addition of copper, which could favors the cost of the production process.

It has been reported for white rot-fungi the production of multiple laccase isoenzymes, which are encoded by different genes and show different expression profiles, stabilities, substrate affinities, and molecular masses (ranging from 35 to 140 kDa) [36,47–49]. Two isoforms have been identified in *T* *. pubescens* strains; two monomeric (Lac1 and Lac2) enzymes of 67 kDa but with different pI (5.3 and 5.1) [50], and two laccase-activity fractions (Lap1 and Lap2) with different pI (> 3.0 and 2.6) from which Lap2 is a monomeric enzyme of 65 kDa [17]. In this study, we identify two laccase isoenzymes from *T* *. pubescens* CBS 696.94 that have different molecular masses (120 and 60 kDa) and pI. The purifications results suggest a more acidic pI for Lac1 (≤ 4) than Lac2 (≥ 6), which differs from a previous report for this fungus, in which two fractions with laccase activity were obtained, Lap1 and Lap2, with pI > 3 and of 2.6, respectively [17]. Since in both cases, similar purification protocols were used, and Lac1 and Lac2 were recovered from the unbound and bound fractions, respectively, as Lap1 and Lap2 [17], this difference could be due to the culture conditions used in each case (i.e. the presence of agro-industrial wastes). Nevertheless, the nanoLC-MS/MS analysis showed that peptides of Lac1 and Lac2 were presented in Lap1 (UniProtKB/Swiss-Prot Q8TG93) and Lap2 (UniProtKB/Swiss-Prot Q8TG94), which have a predicted pI of 4.3 and 4.7, respectively. Lac1 and Lac2 peptides were presented in Laccase-2 (UniProtKB/Swiss-Prot Q99046) and Laccase-1 (UniProtKB/Swiss-Prot Q99044) from *T* *. villosa* , respectively, with 80 to 100% identity. These *T* *. villosa* laccase isoenzymes have a predicted pI of 4.7 (laccase-1) and 6.0 (laccase-2), and a 77% identity among them. In addition, *T* *. villosa* laccase-2 is reported as a homodimer protein, while laccase-1 is reported as monomeric protein. Alignment of Lac1-Lap2 and Lac2-Lap1 from *T* *. pubescens* CBS 696.94 and *T* *. villosa* , showed a 96 and 70% identity, respectively. Finally, similar peptides were identified for Lac1 and Lac2 from *T* *. pubescens* (Schumach.) *Pilát* (*Syn.:* *Coriolus* *pubescens* *(Schum. ex Fr.) Quél.*) BCB 923-2 (Figure 3b) [51]. These results suggest that the purified fractions correspond to laccase isoenzymes that differ from previously reported ones for this *T* *. pubescens* strain.

As mentioned above, laccases isoenzymes present different stabilities and substrate affinities. This behavior was also observed for the laccase isoenzymes purified from *T* *. pubescens* , with Lac1 showing lower laccase activity than Lac2, with ABTS as substrate. However, the difference in activity between these isoenzymes depended on the agro-industrial waste used for the *T* *. pubescens* growth, suggesting a probable effect of the substrate composition on the laccase physicochemical properties. In addition, it was observed that the total laccase activity, presented in the UF retentates, is higher than the laccase activity of the purified fractions, regardless of the agro-industrial byproduct used for the *T* *. pubescens* growth. This suggests a synergistic effect of Lac1 and Lac2, as have been reported for laccases obtained from *Trametes* and *Pleurotus* strains [17,52,53].

The maximum relative activity for the UF retentate was presented at a pH from 2.0 to 4.0, regardless of the agro-industrial byproduct, with a monotonical decrease at pHs above this range. The relation between pH and type of electron donor molecule explains this behavior on laccase activity. It has been reported that when the substrate is an organic hydrogen donor, i.e. phenols, the optimal pH ranges from 3.5 to 6.0 while the laccase activity monotonically decreases from pH 2.5 to 7.0, if the substrate is an electron donor like ABTS [54]. Despite that at different pHs, most of the purified laccase fractions presented a similar performance as the UF retentates, the Lac2 fraction obtained from cedar sawdust differed from the previous observation. For this fraction, the maximum activity was observed at pH 5.0, and despite that at acidic pH (≥ 3.0) the relative activity was higher than 80%, at lower pHs (2.0) it decreased almost in a 50%. Nevertheless, at basic pHs the relative laccase activity presented a sharp decreased that is well correlate with the other laccase fractions. We are not able to attribute this particular trend to the presence of multiple laccase isoenzymes in the fraction since the SDS-PAGE revealed only one band when stained with ABTS, and from the NanoLC-MS/MS analysis no difference in the number of peptides and their sequence was detected. We speculate that this particular behavior can be owed to a compound added in the pre-treatment of the cedar wood that might be used for preserving it that might affect the activity of Lac2 and that was not completely removed during the purification.

Temperature stability evaluation showed that Lac1 is the most thermolabile fraction while the laccase activity reduction in the crude extracts did not seem to be the result of an additive effect of the activity loss of the purified fractions. Although there are no reports of laccase stability under the selected conditions, these results agree with previous reports that state that fungal laccases rapidly decrease their activity at temperatures near 60 °C, and thermal stability correlates with the temperature range of the growth of the source organism [55]. In addition, we previously reported for a laccase crude extract obtained from culturing *T* *. pubescens* on coffee husk an activity loss of 26% and 10% at 60 and 50 °C, respectively, after 8 h at pH 6.0 using syringaldazine as substrate [48]

Finally, an electrochemical characterization was carried out for selected crude extracts and purified fractions. The results showed a difference between the electrochemical performances of extracts and purified fractions with syringaldazine or ABTS. These differences could be owed to the different mechanisms for electron transfer, as discussed above. However, the overall performance of the obtained cyclic voltammograms for ABTS and syringaldazine agrees with results reported by Fernández-Sánchez et al. [25], who used a saturated calomel reference electrode.

## Conclusions

We have shown the potential of coffee husk, soybean pod husk, and cedar sawdust as substrates for the laccase production. In several countries, like Brazil and Colombia, the production of coffee and soybean is of great importance and the generated volume of residues is high. The results showed that semi-solid culture of *T* *. pubescens* using coffee husk allows to obtain an important amount of laccase, which could be favored by the phenolic-compounds, i.e. tannins, presented in coffee husk. While supplementation of the culture medium with copper favored the production of laccase in submerged and semi-solid culture using soybean pod husk and cedar sawdust, semi-solid culture using coffee husk, which presented the highest laccase volumetric yield, did not require copper supplementation. This is an economic advantage that should be considered when scaling up the process. In this study, *T* *. pubescens* showed its ability to produce two independent laccases regardless of the used substrate. However, a higher laccase activity was observed for crude extracts rather than for purified fractions. This suggests that Lac1 and Lac2 might have a synergistic effect on the final laccase activity rather than an additive effect. Based on this study, the investigation of laccase production by evaluating other agro-industrial wastes, in which phenolic-compounds constitute an important percentage of their composition, is of great interest to avoid the use of inducers that would increase the production cost.
